# 

**Published:** 1874-01

**Authors:** 


### CONTENTS:

### COMMUNICATIONS.

Diagnosis . . . . . . . . . . . . . . . . . . . . . . . . . . . . . . 9
Radical Observations about People's Teeth, No II . . . . . . 16
The Deciduous Teeth and their Treatment—Causes of Irregularity of the Permanent Teeth, etc . . . . . . . . . . . . 19
How Much of the Success of Filling Teeth is Due to the Manipulation . . . . . . . . . . . . . . . . . . . . . . . . . . . . 33
Retiring Address . . . . . . . . . . . . . . . . . . . . . . . . . . . 36
Neuralgia Caused by Granules of Osteo Dentine in Pulp Cavities of Teeth . . . . . . . . . . . . . . . . . . . . . . . . . 39
Book Reviews:
The Pathology of the Teeth . . . . . . . . . . . . . . . . . . . . 41
A System of Dental Surgery . . . . . . . . . . . . . . . . . . . . 43

### PROCEEDINGS OF SOCIETIES.

American Academy of Dental Science . . . . . . . . . . . . . . . . 45

### MISCELLANEOUS

The Pulse of Various Animals . . . . . . . . . . . . . . . . . . . 15
Fatal Poisoning from Carbolic Acid . . . . . . . . . . . . . . . . 18

### EDITORIAL.

Found at Last . . . . . . . . . . . . . . . . . . . . . . . . . . . . . 47
Ohio State Dental Society . . . . . . . . . . . . . . . . . . . . . . 49
United States Circuit Court—The Dental Rubber Cases . 51
Palmer's Instruments . . . . . . . . . . . . . . . . . . . . . . . . . 52
Personal . . . . . . . . . . . . . . . . . . . . . . . . . . . . . . . . . 52

